# Robust Antibody Levels in Both Diabetic and Non-Diabetic Individuals After BNT162b2 mRNA COVID-19 Vaccination

**DOI:** 10.3389/fimmu.2021.752233

**Published:** 2021-11-24

**Authors:** Hamad Ali, Abdulmohsen Alterki, Sardar Sindhu, Barrak Alahmad, Maha Hammad, Salman Al-Sabah, Mohammad Alghounaim, Mohammad H. Jamal, Ali Aldei, Mohammad J. Mairza, Maitham Husain, Sriraman Deverajan, Rasheed Ahmad, Preethi Cherian, Irina Alkhairi, Abdullah Alkandari, Jehad Abubaker, Mohamed Abu-Farha, Fahd Al-Mulla

**Affiliations:** ^1^ Department of Medical Laboratory Sciences, Faculty of Allied Health Sciences, Health Sciences Center (HSC), Kuwait University, Jabriya, Kuwait; ^2^ Department of Genetics and Bioinformatics, Dasman Diabetes Institute (DDI), Dasman, Kuwait; ^3^ Department of Otolaryngology, Head, and Neck Surgery, Zain and Al-Sabah Hospitals, Ministry of Health, Kuwait City, Kuwait; ^4^ Medical Division, Dasman Diabetes Institute (DDI), Dasman, Kuwait; ^5^ Department of Immunology and Microbiology, Dasman Diabetes Institute (DDI), Dasman, Kuwait; ^6^ Animal & Imaging Core Facility, Dasman Diabetes Institute (DDI), Dasman, Kuwait; ^7^ Department of Environmental Health, Harvard T.H. Chan School of Public Health, Harvard University, Boston, MA, United States; ^8^ Department of Biochemistry and Molecular Biology, Dasman Diabetes Institute (DDI), Dasman, Kuwait; ^9^ COVID-19 Research Group, Jaber Al-Ahmad Al-Sabah Hospital, Kuwait City, Kuwait; ^10^ Rheumatology Unit, Department of Medicine, Amiri Hospital, Kuwait City, Kuwait; ^11^ Department of Internal Medicine, Amiri Hospital, Kuwait City, Kuwait; ^12^ Planning and Follow-Up Department, Ministry of Health, Kuwait City, Kuwait; ^13^ National Dasman Diabetes Biobank, Dasman Diabetes Institute (DDI), Dasman, Kuwait

**Keywords:** COVID-19, diabetes mellitus type 2, vaccine, mRNA vaccine, BNT162b2, T2D, COVID-19 vaccine, antibodies

## Abstract

The emergence of effective vaccines for COVID-19 has been welcomed by the world with great optimism. Given their increased susceptibility to COVID-19, the question arises whether individuals with type-2 diabetes mellitus (T2DM) and other metabolic conditions can respond effectively to the mRNA-based vaccine. We aimed to evaluate the levels of anti-SARS-CoV-2 IgG and neutralizing antibodies in people with T2DM and/or other metabolic risk factors (hypertension and obesity) compared to those without. This study included 262 people (81 diabetic and 181 non-diabetic persons) that took two doses of BNT162b2 (Pfizer–BioNTech) mRNA vaccine. Both T2DM and non-diabetic individuals had a robust response to vaccination as demonstrated by their high antibody titers. However, both SARS-CoV-2 IgG and neutralizing antibodies titers were lower in people with T2DM. The mean ( ± 1 standard deviation) levels were 154 ± 49.1 *vs.* 138 ± 59.4 BAU/ml for IgG and 87.1 ± 11.6 *vs.* 79.7 ± 19.5% for neutralizing antibodies in individuals without diabetes compared to those with T2DM, respectively. In a multiple linear regression adjusted for individual characteristics, comorbidities, previous COVID-19 infection, and duration since second vaccine dose, diabetics had 13.86 BAU/ml (95% CI: 27.08 to 0.64 BAU/ml, p=0.041) less IgG antibodies and 4.42% (95% CI: 8.53 to 0.32%, p=0.036) fewer neutralizing antibodies than non-diabetics. Hypertension and obesity did not show significant changes in antibody titers. Taken together, both type-2 diabetic and non-diabetic individuals elicited strong immune responses to SARS-CoV-2 BNT162b2 mRNA vaccine; nonetheless, lower levels were seen in people with diabetes. Continuous monitoring of the antibody levels might be a good indicator to guide personalized needs for further booster shots to maintain adaptive immunity. Nonetheless, it is important that people get their COVID-19 vaccination especially people with diabetes.

## Introduction

COVID-19 pandemic has affected people worldwide to unprecedented proportions, even more severely affecting the people with type-2 diabetes mellitus (T2DM) and/or those with other risk factors for developing COVID-19-related complications. To date, more than 190 million people worldwide have been diagnosed with COVID-19, with an overall prevalence rate of at least 2% (1). Although, mortality rates are generally lower (2.2%), the presence of preexisting health conditions such as T2DM, hypertension, cardiovascular disease, and metabolic syndrome may contribute to increased case fatality rates up to 10% (2–4). A myriad of factors may contribute to increased susceptibility of T2DM patients for developing COVID-19 complications, including impaired innate/adaptive immunity, after the onset of a state of chronic, low-grade inflammation called metabolic inflammation. As a result, upon antigen exposure, the obesity-related chronic inflammation may impair macrophage activation and blunt the mechanisms of pro-inflammatory and/or innate cytokine production (5, 6). This altered, obesogenic milieu may partly explain the presence of antiviral-resistance and vaccine escape mechanisms in obese and/or T2DM populations. Moreover, B and T cell responses are compromised in obese and more so in obese T2DM people (7–9). The unfavorable hormone environment also primes for immune response dysregulation (10). Typically, obese T2DM people may have defective innate/adaptive immunity, resulting from the enhanced production of several pro-inflammatory cytokines/chemokines like TNF-α, IFN-γ, IL-1β, IL-12, IL-18, RANTES, MCP-1, and IL-6 (11). Indeed, all these bioactive inflammatory proteins are also the factors that have been associated with an increased susceptibility for developing COVID-19 complications (12, 13).

Driven by sheer urgency, COVID-19 vaccines have been developed at a phenomenal speed. One of the most widely used vaccines is known as BNT162b2 (Pfizer–BioNTech), which is an mRNA-based COVID-19 vaccine that comprises of the nucleoside-modified mRNA (modRNA sequence of 4,284 nucleotides) encoding a mutated form (bases 103-3879) of the full-length spike (S) protein (peplomer) of SARS-CoV-2 stabilized in its prefusion conformation as an antigen or immunogenic molecule encapsulated in lipid nanoparticles that act as adjuvants (14). The vaccine is administered as two shots, intramuscularly, given 3 weeks apart, and has been shown to offer protection by triggering an immune response against infection by the SARS-CoV-2 spike protein. Since BNT162b2 vaccine delivers mRNA encoding only for SARS-CoV-2 spike protein, the expected elicited response is production of anti-S-RBD immunoglobulin G (IgG), IgM, and IgA isotypes, with neutralization potential of inhibiting the RBD binding to ACE2 cognate receptor (15). As T2DM patients are at higher risk of severe COVID-19 symptoms and mortality, such patients have been prioritized to receive COVID-19 vaccinations (16).

This study aimed to evaluate the humoral immune responses in people with and without T2DM and/or other metabolic risk factors, such as hypertension and obesity. Herein, we present data showing levels of SARS-COV-2-specific IgG as well as anti-S-RBD neutralizing antibodies in a population with a high T2DM prevalence.

## Methods

### Recruitment of Participants and Study Cohort

This study was reviewed and approved by the Ethical Review Committee of Dasman Diabetes Institute “Protocol # RA HM-2021-008” as per the updated guidelines of the Declaration of Helsinki (64th WMA General Assembly, Fortaleza, Brazil, October 2013) and of the US Federal Policy for the Protection of Human Subjects. The study was also approved by the Kuwait Ministry of Health ethical committee (reference: 3799, protocol number 1729/2021). This study aimed at recruiting people that passed a minimum of 3 weeks after taking the second dose of BNT162b2 (Pfizer–BioNTech) mRNA vaccine. People with autoimmune diseases, those taking immunosuppressants or suffering from arthritis were excluded from participating in this study as well as people with Type 1 Diabetes and pregnant women. T2DM diagnosis was based on self-reporting. Data for each participant were captured using a RedCap survey that included age, gender, existing diseases (e.g., diabetes and hypertension), as well as height, weight, and history of COVID-19 infection. Obesity was defined according to body mass index (BMI); those with BMI < 25 kg/m^2^ were considered normal weight, those with BMI between 25 and 30 kg/m^2^ considered overweight, and those with BMI > 30 kg/m^2^ were considered obese. Participants were then asked to visit the Dasman Diabetes Institute where they signed up the informed consent form before participating in the study.

### Blood Sample Collection and Processing

After signing the consent form, a venous blood sample was collected in Vacutainer EDTA tubes. The blood was then centrifuged at 400 × g for 10 min at room temperature to isolate plasma. Plasma samples were then aliquoted and stored at −80°C until the assays were performed.

### Measurement of Plasma Levels of SARS-CoV-2-Specific IgG

Plasma levels of SARS-CoV-2-specific IgG antibodies were detected using enzyme-linked immunosorbent assay (ELISA) kit (SERION ELISA agile SARS-CoV-2 IgG SERION Diagnostics, Würzburg, Germany), following the manufacturer’s instructions. Briefly, plasma samples were thawed at room temperature and centrifuged for 5 min at 10,000 ×g at 4°C for sample clarification and removal of residual cells or platelets. For assay, samples were diluted 1:100 using dilution buffer (phosphate buffer with tween 20). Then, 100 µl each of diluted samples, negative control, positive control, and standard sera were transferred into the designated wells in triplicate and incubated for 60 min at 37°C in a humid chamber. Following incubation, plates were washed four times with 1× wash buffer (physiological saline with tween 20 and 30 mM Tris/HCl; 300 µl/well), and later, anti-human IgG polyclonal antibody enzyme conjugate was added (100 µl/well). The plates were then incubated for 30 min at 37°C in humid chamber and later washed four times as before. Next, 100 µl of chromogenic substrate (para-nitrophenyl phosphate in solvent-free buffer containing 0.1% sodium azide) was added into each well, and plates were incubated for 30 min at 37°C under humidity in the dark. Finally, the reaction was stopped using the stop solution (0.1 N NaOH, 40 mM EDTA), plates were gently shaken to mix, and absorbance was read (Synergy H5 plate reader) within 60 min to measure optical density (O.D.) at 405 nm wavelength, against substrate blank wavelength at 650 nm. Antibody quantification by mathematical curve fitting was based on the four-parameter logistic (4 PL) function as below:


Concentration=exp [4.187−ln{2.78/(OD Sample×0.85/OD Standard−0.012)−1{/1.088}]

The test evaluation was performed following the positive and negative cutoffs as recommended by the manufacturer, and IgG levels were reported as binding antibody units (BAU)/ml. In this regard, IgG levels of <21 BAU/ml were considered negative, levels of 21.0–31.5 BAU/ml were taken as borderline, and levels higher than 31.5 BAU/ml were considered as positive. The sensitivity and specificity of the kit were 96.2 and 99.2%, respectively.

### Measurement of Plasma Levels of SARS-CoV-2-Specific Neutralizing Antibody

SARS-CoV-2-specific surrogate Virus Neutralization Test (sVNT) was used to detect levels of plasma neutralizing antibodies against SARS-CoV-2 S-RBD (SARS-Cov-2 sVNT kit, GenScript, USA, Inc). For sVNT assay, briefly, clarified plasma samples and positive and negative controls were diluted 1:10 using sample dilution buffer provided in the kit. Horseradish peroxidase (HRP)-conjugated recombinant SARS-CoV-2 receptor binding domain (HRP-RBD) was diluted 1:1,000 using HRP dilution buffer. Wash solution was prepared by diluting 1:20 with deionized water. Capture plate was prepared to run each of test samples and positive and negative controls in duplicate. In separate tubes, diluted test samples and positive/negative controls were mixed with diluted HRP-RBD solution in 1:1 volume ratio and incubated at 37°C for 30 min. Later, test samples and positive/negative controls, 100 μl each, were transferred into designated wells in the plate and incubated for 15 min at 37°C. After incubation, wells were washed four times with 1× wash buffer (260 μl/well), and then TMB substrate solution (100 μl) was added into each well and plates were incubated in the dark for 15 min at 20–25°C. Finally, the reaction was quenched by adding 50 μl of stop solution to each well, and O.D. was measured at 450 nm wavelength using Synergy H5 plate reader. The test evaluation was carried out following the recommended positive and negative cutoffs, and test results were interpreted by calculating inhibition rates for samples as follows:


Inhibition=(1−O.D. value of sample/O.D. value of negative control)×100%

According to the manufacturer’s instructions, neutralizing antibody levels higher than 20% were considered as positive. The intra-assay and inter-assay variations of the kit were reported as ≤10 and ≤15%, respectively.

### Statistical Analysis

We provide summary descriptive analysis using mean, median, standard deviation, and interquartile range as appropriate. We fitted generalized additive linear models with plasma IgG levels and neutralizing antibodies as the dependent variables and T2DM status (yes/no), hypertension (yes/no), BMI (categorical: <25, 25–30, and >30 kg/m^2^), age (linear), gender, comorbidity score (sum score of equal weight for heart disease, stroke, chronic obstructive pulmonary disease, asthma, obstructive sleep apnea, chronic kidney disease, bleeding disorders, and other chronic diseases), and duration since receiving the second vaccine dose as the independent variables. We fitted penalized splines for the duration since second dose to account for non-linearity using restricted maximum likelihood (REML) estimation. Penalized splines are smoothing non-parametric terms that can maximize the goodness-of-fit by cross-validation and a penalty term for over- and underfitting. We reported effect estimates with 95% confidence intervals interpreted as change in average IgG and neutralizing levels after adjustment.

In an additional *post-hoc* analysis, we also explored potential effect measure modifiers on the relationship between diabetes and IgG levels after vaccination. The effect measure modification models were selected based on the same model used in the main analysis. We considered interactions by age, gender, BMI, and hypertension categories. In each stratum of age, gender, BMI, and hypertension, we estimated the change in IgG levels comparing diabetics to non-diabetics. The p-value for interaction was obtained from the Wald-test of the interaction term coefficient. All analyses were done in R software version 3.3.1 (R Foundation for Statistical Computing), and penalized splines were implemented in generalized additive models using the *mgcv* package. A p-value of less than 0.05 was considered to indicate statistical significance.

## Results

### Population Characteristics

This study included a total of 262 participants: 181 non-diabetics and 81 people diagnosed with T2DM. Of these 262 individuals, 193 were normotensive and 69 were hypertensive. Subdividing based on body mass index (BMI), 65 people had a BMI less than 25 kg/m^2^(normal weight), 117 people had a BMI between 25 and 30 kg/m^2^ (overweight), and 74 had a BMI higher than 30 kg/m^2^ (obese); BMI information for six individuals was not available. This cohort included 126 females and 136 males **(** **Table 1** **)**.

**Table 1:** Clinical characteristics and SARS-CoV2 serological findings in studied groups stratified by type-2 diabetes mellitus status.

	Non-diabetics	Diabetics	Overall
	(N=181)	(N=81)	(N=262)
**Age (years)**			
Mean (SD)	44.8 (13.2)	59.4 (12.0)	49.3 (14.5)
Median (Min, Max)	43.1 [21.0, 87.4]	60.3 [24.4, 81.2]	50.3 [21.0, 87.4]
**Gender**			
Female	92 (50.8%)	34 (42.0%)	126 (48.1%)
Male	89 (49.2%)	47 (58.0%)	136 (51.9%)
**BMI categories**			
Less than 25	51 (28.2%)	14 (17.3%)	65 (24.8%)
Between 25 and 30	83 (45.9%)	34 (42.0%)	117 (44.7%)
Greater than 30	43 (23.8%)	31 (38.3%)	74 (28.2%)
Missing	4 (2.2%)	2 (2.5%)	6 (2.3%)
**Hypertension**			
No	151 (83.4%)	42 (51.9%)	193 (73.7%)
Yes	30 (16.6%)	39 (48.1%)	69 (26.3%)
**Comorbidity score**			
Mean (SD)	0.320 (0.594)	0.469 (0.896)	0.366 (0.703)
Median (Min, Max)	0 [0, 3.00]	0 [0, 5.00]	0 [0, 5.00]
**Previous infection of COVID 19**			
No	155 (85.6%)	69 (85.2%)	224 (85.5%)
Yes	26 (14.4%)	12 (14.8%)	38 (14.5%)
**Duration since second dose**			
Mean (SD)	84.3 (37.1)	81.5 (37.4)	83.4 (37.2)
Median (Min, Max)	82.0 [7.00, 148]	81.0 [13.0, 148]	82.0 [7.00, 148]
**IgG (BAU/ml)**			
Mean (SD)	154 (49.1)	138 (59.4)	149 (52.9)
Median (Min, Max)	160 [11.0, 243]	140 [19.3, 264]	156 [11.0, 264]
**IgM (BAU/ml)**			
Mean (SD)	65.6 (84.2)	58.1 (112)	63.3 (93.6)
Median (Min, Max)(	31.3 [0.600, 600]	30.5 [0, 800]	31.0 [0, 800]
**Neutralizing antibodies (%)**			
Mean (SD)	87.1 (11.6)	79.7 (19.5)	84.8 (14.9)
Median (Min, Max)	91.5 [22.6, 95.3]	88.0 [0, 95.8]	90.9 [0, 95.8]
Missing	3 (1.7%)	0 (0%)	3 (1.1%)

### SARS-CoV-2 IgG and Neutralizing Antibodies in People With or Without T2DM

**Figure 1:**
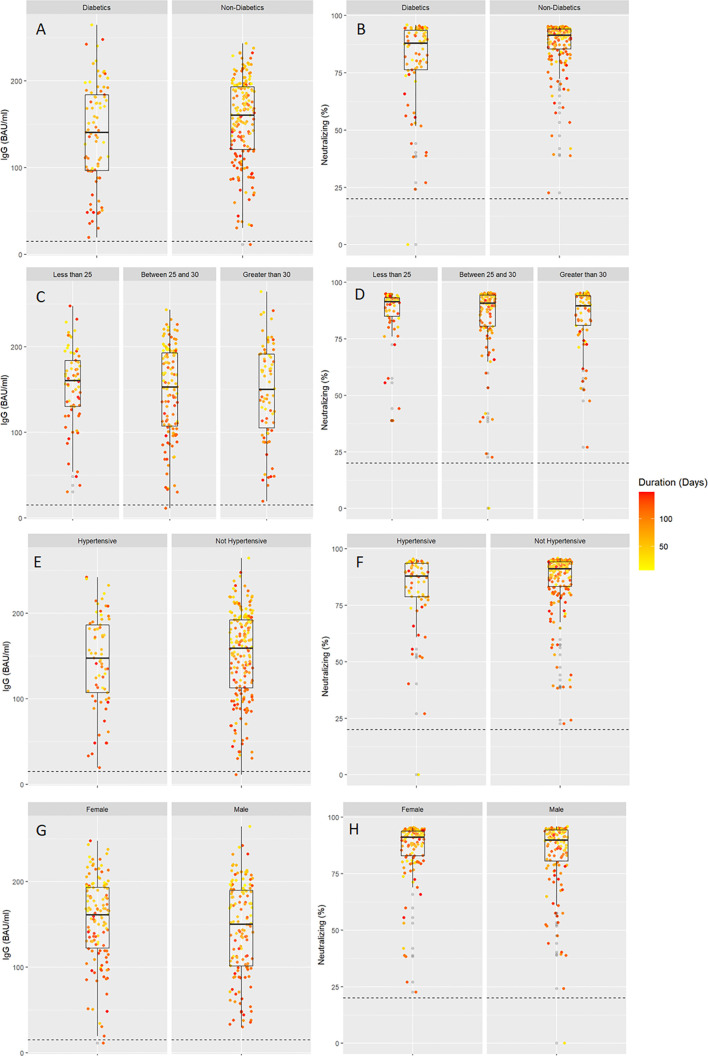
SARS-CoV2 IgG and neutralizing antibodies in individuals stratified by diabetes **(A, B)**, obesity levels **(C, D)**, hypertension **(E, F)**, and gender **(G, H)**. All individuals took two doses of BNT162b2 (Pfizer–BioNTech) vaccine, and this was plotted with days since vaccination shown based on the color intensity.

Analysis of the SARS-CoV-2 IgG and neutralizing antibodies based on T2DM status showed that all people elicited robust levels of these antibodies. Overall, SARS-CoV-2 IgG level was 149 ± 52.9 BAU/ml, which was more than five times the cutoff for a positive level as recommended by the manufacturer. However, stratifying our cohort based on the T2DM status, people with T2DM had lower levels of SARS-CoV-2 IgG antibodies (138 ± 59.4 BAU/ml) compared to people without diabetes (154 ± 49.1 BAU/ml) (**Figure 1A**). Similarly, strong SARS-CoV-2 neutralizing antibody levels were overall observed in our study cohort; however, neutralizing antibody levels were also lower in people with T2DM (79.7 ± 19.5%) compared to those without T2DM (87.1 ± 11.6%) (**Figure 1B**). Clinical characteristics and serological findings in the studied population stratified by T2DM status are summarized in (**Table 1** **)**.

After adjustment to potential confounders, on average, people with TD2M had 13.86 BAU/ml (95% CI: 27.08 to 0.64 BAU/ml, p=0.041) less IgG antibodies and 4.42% (95% CI: 8.53 to 0.32%, p=0.036) less neutralizing antibodies than non-diabetics (**Table 2**).

**Table 2:** Multiple linear regression analyses showing average changes of SARS-CoV2 IgG and neutralizing antibodies.

Variable	IgG (BAU/ml)*			Neutralizing (%)*		
	Change	95% CI	p-value	Change	95% CI	p-value
Diabetic *vs.* Non-Diabetic	−13.86	[−27.08 to −0.64]	0.041	−4.42	[−8.53 to −0.32]	0.036
Hypertensive *vs.* Not Hypertensive	4.00	[−9.95 to 17.95]	0.575	0.75	[−3.59 to 5.1]	0.734
Age (per 1 year increase)	−0.43	[−0.86 to 0]	0.049	−0.25	[−0.38 to -0.12]	<0.001
Male *vs.* Female	−3.52	[−15 to 7.96]	0.548	−0.31	[−3.87 to 3.26]	0.865
BMI between 25 and 30 *vs.* BMI < 25	−8.70	[−22.58 to 5.19]	0.221	−1.91	[−6.29 to 2.46]	0.392
BMI Greater than 30 *vs.* BMI < 25	−5.76	[−21.57 to 10.05]	0.476	−0.33	[−5.3 to 4.63]	0.895
Previous COVID-19 Infection *vs.* None	38.50	[23.05 to 53.96]	<0.001	7.11	[2.31 to 11.91]	0.004
Comorbidity score (per 1 score increase)	4.99	[−3.12 to 13.1]	0.229	1.21	[−1.35 to 3.77]	0.354

*All models were adjusted for the variables mentioned in addition to duration since second dose of BNT162b2 (Pfizer–BioNTech) vaccine, which was modeled non-linearly with penalized splines in generalized additive models.

### SARS-CoV-2 IgG and Neutralizing Antibodies and Obesity Levels

SARS-CoV2 IgG antibody levels in people with normal weight, overweight, and obesity were 153 ± 47.6, 147.0 ± 53.4, and 148.0 ± 56.7 BAU/ml, respectively (**Figure 1C**). Similarly, SARS-CoV2 neutralizing antibody levels were also comparable in our study population, and the levels in people with normal weight, overweight, and obesity were 86.3 ± 12.8, 84.3 ± 16.6, and 84.6 ± 13.6%, respectively (**Figure 1D**). Obesity status did not show a statistically significant difference in regression analyses (**Table 2**).

### SARS-CoV-2 IgG and Neutralizing Antibodies in People With or Without Hypertension

Next, we found that the circulating levels of SARS-CoV2 IgG antibodies were comparable between people with or without hypertension. In people with hypertension, SARS-CoV2 IgG antibody levels were relatively lower (144 ± 54.8 BAU/ml) compared to those without hypertension (151 ± 52.2 BAU/ml) (**Figure 1E**). Similarly, neutralizing antibody levels also differed between people with hypertension and those without hypertension (81.7 ± 17.3 *vs* 85.9 ± 13.8%) (**Figure 1F**). After adjustment to potential confounders, the differences in humoral immunity by hypertension status were not statistically significant (p=0.575 for IgG, and p=0.734 for neutralizing antibodies) (**Table 2**).

### SARS-CoV-2 IgG and Neutralizing Antibodies by Gender and Age

Circulating levels of SARS-CoV2 IgG antibodies were not significantly different when compared between male and female (p=0.548) (**Table 2**). Overall, females had a higher SARS-CoV2 IgG antibody level of 154.0 ± 50.0 BAU/ml compared to 144.0 ± 55.2 BAU/ml in male (**Figure 1G**). Similarly, neutralizing antibody levels were not statistically significant (p=0.865) (**Table 2**) between female and male, which were 85.9 ± 13.5 *vs* 83.8± 16.0%, respectively (**Figure 1H**). Regarding participant age, each 1 year increase in age was associated with −0.43 BAU/ml (95% CI: −0.86 to 0, p=0.049) and −0.25% decrease in neutralizing antibodies (95% CI: −0.38 to −0.12%, p<0.001) **(** **Table 2** **)**.

### Additional Analyses

**Figure 2:**
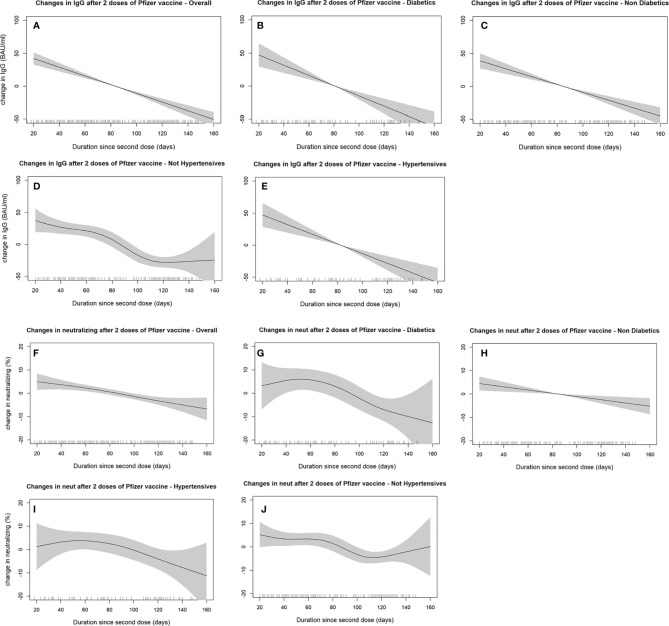
SARS-CoV2 IgG **(A–E)** and neutralizing antibodies **(F–J)** decline over time since receiving the second dose of BNT162b2 (Pfizer–BioNTech) vaccine stratified by diabetes and hypertension status. The smooth relationships were derived from generalized additive models with penalized splines for duration (in days) and adjusted for age, gender, BMI, hypertension, diabetes status, comorbidity scores, and previous COVID-19 infections. Solid lines represent the effect estimates of change over time, while the shaded areas represent the 95% confidence intervals.

Generally, IgG and neutralizing antibody levels declined as more days passed after receiving the second vaccine dose. Antibodies-duration adjusted smooth relationships with 95% confidence intervals are presented in (**Figure 2** **)**. The best fitting smoothed relationship was linear with only one effective degree of freedom, as determined by the penalized spline. Diabetes and hypertensive status did not show considerable deviation from the linear decline over time, nor did we notice any appreciable change in slope between different subgroups. Regression estimates using IgM antibodies as the outcome also did not show any significant differences between diabetics and non-diabetics (**Supplemental Table S1**). In the interaction analyses, across all groups, being diabetic was consistently associated with lower IgG levels as compared to non-diabetics (**Supplemental Figure S1**). However, the interaction terms between diabetes status and the other variables (age, gender, previous COVID-19 infection, BMI, and hypertension) were not powered to result in statistically significant p-values.

## Discussion

In the present study, SARS-CoV-2-specific IgG and neutralizing antibody responses were evaluated in a cohort of 262 adult individuals aged 49 ± 15.1 years, following two doses of BNT162b2 (Pfizer-BioNTech) mRNA vaccine and analyzed regarding diabetic status, hypertension, BMI, gender, and duration after second dose. Our data show that robust levels of IgG and neutralizing antibodies, nearly five times the seropositivity threshold, were elicited after immunization. Whereas, all antibody isotypes can mediate SARS-CoV-2 neutralization (17–19), IgG is the major antibody isotype found in blood and extracellular fluid (20) and can inhibit infectivity of SARS-CoV-2 by directly blocking viral attachment to ACE2 on host cell to preventing viral entry (21, 22). Notably, BNT162b2 vaccine delivers mRNA encoding SARS-CoV-2 S-glycoprotein, and it induces only the production of anti-S-RBD and not anti-N IgG antibodies (23). Increased anti-S-RBD IgG antibody titers indicate seroconversion after either disease exposure or immunization (24). Robust SARS-CoV-2 IgG and neutralizing antibody levels in our study cohort after two doses of BNT162b2 mRNA vaccine suggest proficient antigen processing and presentation in all individuals as indicated by other BNT162b2 vaccination studies in diverse cohorts (15, 25–27).

Detecting high titers of anti-RBD IgG antibodies does not reflect their functional significance in immunoprotection. There is a strong positive correlation reported between virus neutralization assays and anti-RBD antibodies-mediated interaction inhibition between SARS-CoV-2 spike (S1) protein and ACE2 receptor (28). Therefore, sVNT was used and high levels of neutralizing antibodies were detected, indicating that BNT162b2 mRNA vaccine induced protective immunity in these individuals. In line with this, Pratesi et al. showed that BNT162b2 mRNA vaccine induced increased levels of high-avidity, anti-RBD IgG/IgA, and protective neutralizing antibodies in the vaccinated cohort (15).

We further assessed whether presence of T2DM impacted the humoral immune response proficiency in response to BNT162b2 mRNA vaccine. To this end, the data showing increased levels of anti-RBD IgG and neutralizing antibodies in T2DM cohort are consistent, at least in part, with the study reporting that humoral immune response against SARS-CoV-2 in patients with diabetes, regarding timing and antibody titers, was comparable to that of non-diabetic patients (29). In our study, significantly lower levels of IgG and neutralizing antibodies in T2DM cohort are comprehensible since hyperglycemia and insulin resistance are known to induce immune defects, such as impairment in monocyte/macrophage and neutrophil function, reduced lymphocyte proliferation, defective antigen presentation, and complement dysfunction (30). The lower humoral responses in our T2DM cohort are in line with the study by Yelin et al. reporting similar effectiveness of BNT162b2 across age groups but lower vaccine efficacy in people with chronic comorbidities, including T2DM (31). There are growing speculations that humoral immune response to COVID-19 might be inadequate in people with T2DM and hence they might be at a high risk of reinfections with COVID-19 (8, 9). Of note, the people with type-1/type-2 diabetes may have to be prioritized for vaccination, given their high risk of poor prognosis with COVID-19 (16). SARS-CoV-2 IgG and neutralizing antibody levels did not differ significantly in our study cohort with regard to BMI, age, and gender. In partial agreement to this, a study of antibody titers at 7 days after second dose of BNT162b2 vaccine reported that age and gender significantly impacted humoral immune responses, but BMI and hypertension did not (32). The disparities among outcomes of BNT162b2 studies regarding effects of age, gender, BMI, hypertension, and diabetes status on immune responses may relate to differences with respect to cohorts, time after second vaccination, comorbidities, and medication.

Nonetheless, this study is limited by certain caveats, which warrant caution. First, the study population was self-selected, mostly by word of mouth and recruitment advertisements. It is likely that the sample over-represented comorbid individuals that actively sought out for knowing their immunoprotection after vaccination. Second, the regression analyses were adjusted for a number of *a priori* confounders; however, we cannot rule out potential residual confounding by severity of illness. To this end, we could not assess whether patients with severe uncontrolled diabetes or hypertension showed relatively lower humoral responses to vaccination. Third, small sample size also limited the ability to examine possible differential effects among subgroups, and interaction analyses were likely underpowered to detect significant differences between different strata. Fourth, the data presented represent a cross-sectional analysis of only the humoral response; therefore, further studies will be required for longitudinal analyses of both arms of adaptive immunity to BNT162b2 vaccination, with longer follow-ups to detect the duration of protective immunity. Finally, the studied population might be healthier than the general population in Kuwait and is likely to be homogenous. The readers should be cautioned when generalizing findings to other settings and populations. Future research should be performed involving larger-cohort, multicenter studies analyzing protective immunity as well as presence of memory immune cell populations.

## Conclusion

Taken together, our data support the presence of robust SARS-CoV-2-specific IgG and neutralizing antibody responses in people with and without T2DM, following two doses of Pfizer-BioNTech BNT162b2 mRNA vaccine. Notwithstanding, the T2DM cohort had significantly lower antibody titers than non-diabetics, whereas age, gender, BMI, and hypertension did not show significant effect on antibody titers. Importantly, continuous monitoring of SARS-CoV-2-specific IgG and neutralizing antibody profiling may be a pragmatic approach to guide the personalized needs, especially of those with T2DM, for booster shots to maintain protective immunity in COVID-19 vaccinees.

## Data Availability Statement

The raw data supporting the conclusions of this article will be made available by the authors upon request.

## Ethics Statement

This study was reviewed and approved by the Ethical Review Committee of Dasman Diabetes Institute “Protocol # RA HM-2021-008” as per the updated guidelines of the Declaration of Helsinki (64th WMA General Assembly, Fortaleza, Brazil, October 2013) and of the US Federal Policy for the Protection of Human Subjects. The study was also approved by the Kuwait Ministry of Health ethical committee (reference: 3799, protocol number 1729/2021). The patients/participants provided their written informed consent to participate in this study.

## Author Contributions

HA, AT, and SS conceived and designed the analysis, researched data, and wrote the manuscript. BA performed the analysis and generated the figures. MHa was involved in sample collection and laboratory analysis. SS, MG, MJ, AlA, AbA, MM, and MHu were involved in subjects’ recruitment and reviewed/edited the manuscript. IA and PC performed laboratory analysis. SD was involved in data collection and managed recruitment. RA edited the manuscript. JA, MA-F, and FA contributed to the discussion and reviewed/edited the manuscript. All authors contributed to the article and approved the submitted version. MA-F is the guarantor of this work and, as such, had full access to all the data in the study and takes responsibility for the integrity of the data and the accuracy of the data analysis.

## Funding

This Study was funded by Kuwait Foundation for the Advancement of Sciences (KFAS) grant (RA HM-2021-008).

## Conflict of Interest

The authors declare that the research was conducted in the absence of any commercial or financial relationships that could be construed as a potential conflict of interest.

## Publisher’s Note

All claims expressed in this article are solely those of the authors and do not necessarily represent those of their affiliated organizations, or those of the publisher, the editors and the reviewers. Any product that may be evaluated in this article, or claim that may be made by its manufacturer, is not guaranteed or endorsed by the publisher.
